# The Co-Transplantation of Bone Marrow Derived Mesenchymal Stem Cells Reduced Inflammation in Intramuscular Islet Transplantation

**DOI:** 10.1371/journal.pone.0117561

**Published:** 2015-02-13

**Authors:** Gumpei Yoshimatsu, Naoaki Sakata, Haruyuki Tsuchiya, Takashi Minowa, Taro Takemura, Hiromi Morita, Tatsuo Hata, Masahiko Fukase, Takeshi Aoki, Masaharu Ishida, Fuyuhiko Motoi, Takeshi Naitoh, Yu Katayose, Shinichi Egawa, Michiaki Unno

**Affiliations:** 1 Department of Surgery, Tohoku University Graduate School of Medicine, Sendai, Japan; 2 Nanotechnology Innovation Station, National Institute for Materials Science, Tsukuba, Japan; 3 Division of Integrated Surgery and Oncology, Tohoku University Hospital, Sendai, Japan; 4 Division of International Cooperation for Disaster Medicine, Tohoku University, Sendai, Japan; Children’s Hospital Boston/Harvard Medical School, UNITED STATES

## Abstract

**Aims/Hypothesis:**

Although the muscle is one of the preferable transplant sites in islet transplantation, its transplant efficacy is poor. Here we attempted to determine whether an intramuscular co-transplantation of mesenchymal stem cells (MSCs) could improve the outcome.

**Methods:**

We co-cultured murine islets with MSCs and then analyzed the morphological changes, viability, insulin-releasing function (represented by the stimulation index), and gene expression of the islets. We also transplanted 500 islets intramuscularly with or without 5 × 10^5^ MSCs to diabetic mice and measured their blood glucose level, the glucose changes in an intraperitoneal glucose tolerance test, and the plasma IL-6 level. Inflammation, apoptosis, and neovascularization in the transplantation site were evaluated histologically.

**Results:**

The destruction of islets tended to be prevented by co-culture with MSCs. The stimulation index was significantly higher in islets co-cultured with MSCs (1.78 ± 0.59 vs. 7.08 ± 2.53; p = 0.0025). In terms of gene expression, *Sult1c2*, *Gstm1*, and *Rab37* were significantly upregulated in islets co-cultured with MSCs. Although MSCs were effective in the *in vitro* assays, they were only partially effective in facilitating intramuscular islet transplantation. Co-transplanted MSCs prevented an early inflammatory reaction from the islets (plasma IL-6; p = 0.0002, neutrophil infiltration; p = 0.016 inflammatory area; p = 0.021), but could not promote neovascularization in the muscle, resulting in the failure of many intramuscular transplanted islets to engraft.

**Conclusions:**

In conclusion, co-culturing and co-transplanting MSCs is potentially useful in islet transplantation, especially in terms of anti-inflammation, but further augmentation for an anti-apoptosis effect and neovascularization is necessary.

## Introduction

Islet transplantation is a promising treatment for insulin-dependent diabetes mellitus (DM). In the clinic, islets are usually transplanted into the liver; this strategy is based on the success of a preclinical animal study [1]. However, the liver is not an ideal transplant site [2] because many transplanted islets are damaged due to the instant blood-mediated inflammatory reaction (IBMIR) [3], a nonspecific inflammatory reaction caused by Kupffer cells [4] and natural killer T cells [5], lipotoxicity [6,7], or ischemia caused by embolization of the peripheral portal vein [8,9]. In addition, intraportal transplantation is associated with an increased risk of portal embolization and portal hypertension, which could sometimes become severe [10,11]. According to the Fiorina’s review, rate of acute complications is 2% to 3% for hemorrhage and 3% for partial portal vein thrombosis [12].

Various organs have been studied as an alternative transplantation site, including the kidney [13], greater omentum [14], bone marrow [15], pancreas [16], and eye [17]. However, most of these transplantation sites are suboptimal in a clinical setting, as they require a special technique or invasive procedure. Therefore, in the present study we focused on intramuscular islet transplantation because it is technically easy and safe, and it does not cause severe complications. Even if some predictable complications such as bleeding, hematoma and infection [2] may occur, it is relatively easy to control them. However, the efficacy of the intramuscular transplant was reported to be inferior to intraportal transplantation [18,19]. A previous report described that the causes of poor transplant efficacy were poor oxygen tension and blood supply from the lack of early neovascularization [20]. Thus, preventing the early loss of transplanted islets in the muscle is necessary for improving the transplant efficacy.

Mesenchymal stem cells (MSCs) are adult progenitor cells, representing 0.001%–0.01% of the bone marrow population [21], and they have many unique functions including pluripotency, self-proliferation, and trophic effects such as tissue repair, the reduction of inflammation and apoptosis, and the promotion of neovascularization [22–24]. MSCs may contribute to the improvement of the engraftment of co-transplanted cells. Actually, our previous study clarified that syngeneic co-transplanted bone marrow cells (including MSCs) improved the outcome of islet transplantation into the renal subcapsule by promoting neovascularization [25]. Park and colleagues also performed similar trials using human MSCs, and showed improvement of the islet transplant effect with enhanced blood vessel formation [26]. Ito and colleagues also proved the usefulness of MSC co-transplantation using an intraportal islet transplant rodent model [23]. We hypothesized that MSC co-transplantation into the intramuscular space also could improve the function and engraftment of transplanted islets by ‘trophic effects’.

We tested this hypothesis and analyzed the *in vitro* effects on the gene expression of islets in this study.

## Materials and Methods

### Ethics

All animal care and treatment procedures were carried out in accord with the Regulations for Animal Experiments and Related Activities at Tohoku University, and the protocol was approved by the Institutional Animal Care and Use Committee of Tohoku University Graduate School of Medicine (The approved protocol number is 2013 IDO-112).

### Animals

Syngenic BALB/c mice (9 to 12-wk-old males; CLEA Japan, Tokyo) were used as recipients and donors of islets and MSCs for transplantation. The animals were housed under pathogen-free conditions on a 12-h light cycle with free access to food and water.

### Isolation of MSCs

MSCs were isolated from bone marrow cells of BALB/c mice as described [27]. The acquired bone marrow cells were cultured in MSC culture medium consisting of Eagle’s minimum essential medium (E-MEM; Sigma-Aldrich, St. Louis, MO) supplemented with 20% fetal bovine serum (FBS; Hyclone Laboratories, Logan, UT), 2 mM L-glutamine (Life Technologies, Carlsbad, CA), and 1% antibiotics (PenStrep, Life Technologies). The cultured media were changed 3 d after starting the culture, and every second day thereafter. Floating hematopoietic cells were removed by changing the media, and MSCs were proliferated. Confluent MSCs at primary culture were used for this study after detachment with 0.25% trypsin-ethylenediaminetetraacetic acid (EDTA, Sigma-Aldrich).

### Characterization of MSCs

Pluripotent potential and expression of specific surface markers were identified. We validated pluripotency as an osteogenic/adipogenic differentiation assay by using Osteoblast-Inducer Reagent and Adipo-Inducer Reagent (Takara Biotechnology, Shiga, Japan; S1 Fig.). The surface markers of MSCs were stained; positive for CD29, CD44, CD73, CD105, CD106, and Sca-1 and negative for CD11b and CD45. All of the primary antibodies (Mouse Mesenchymal Stem Cell Marker Antibody Panel; R&D Systems, Spokane, WA) were diluted 1:100 in phosphate buffered saline (PBS) supplemented with 1% bovine serum albumin and 0.3% Triton X-100 (Wako Pure Chemical Industries, Osaka, Japan). Cultured MSCs were respectively incubated with primary antibody solutions overnight at 4°C. Thereafter, they were incubated with a fluorophore-conjugated secondary antibody (Alexa Fluor488 conjugate anti-rat IgG antibody; Cell Signaling Technology, Danvers, MA or FITC-labeled rabbit anti-sheep IgG antibody, Pierce Biotechnology, Rockford, IL) at room temperature for 2 h. After counterstaining with DAPI (Vector Laboratories, Burlingame, CA), they were visualized using a BZ-9000 fluorescent microscope (Keyence Japan, Osaka, Japan).

### Islet isolation

The protocol of islet isolation was a modification of Gotoh’s method [28]. The pancreata were expanded by an injection of collagenase solution (Collagenase type V; Sigma-Aldrich). They were digested, and purified by discontinuous Ficoll (Ficoll PM 400; Sigma-Aldrich) gradient centrifugation and by handpicking. They were used after overnight culture in the culture medium: RPMI 1640 (Sigma-Aldrich) with 5.5mM glucose, 10% FBS and 1% PenStrep.

### *In vitro* assessments

Fifty islets were handpicked and cultured in a 24-well plate and designated as Group A. In Group B, 5×10^4^ MSCs were added to the islets. Various islet parameters were compared between group A (cultured without MSCs) and group B (co-cultured with MSCs).

1. Morphological change of cultured islets was observed in the culture medium at 4-d culture.
2. Viability: Islets were stained with SYTO 11 Green (Life Technologies; viable cells at green) and ethidium bromide (Sigma-Aldrich; dead cells at red) at 1 and 4 d after the culture. Using the image under a confocal microscope (C2Si, Nikon, Tokyo), the viability was calculated by Image J software v.1.47 (National Institutes of Health; MD, USA) as follows: viability rate of islets (%) = (green area / (green area + red area)) × 100
3. Insulin releasing function: The glucose response of the islets was estimated by static incubation at 1 and 4 d after the culture [29]. Ten islets from each group were handpicked. After the 1-h pre-incubation in low-glucose medium (3.3 mM), they were further incubated in fresh low-glucose medium for an additional 1 h to collect the supernatants (sample L). The islets were then cultured in fresh high-glucose medium (16.5 mM) for 1 h and the supernatants were collected (sample H). The insulin volume was measured using a Mouse ELISA Insulin Kit (Shibayagi Co., Gunma, Japan). We used the stimulation index, which was defined as the ratio of insulin volume between samples H and L, for evaluation of insulin releasing function. It was considered that the islets with high (over 1) stimulation index were good in insulin-releasing function depended on the glucose concentration.

### Comprehensive gene expression analysis of islets with or without MSCs

We used a DNA microarray to conduct a comprehensive gene expression analysis of the islets. Islets cultured for 24 h were classified into four samples by treatment: Sample 1 was islets only (S1) as the control. Sample 2 was islets co-cultured with syngenic MSCs but without cell-to-cell contact using a Transwell (Corning, New York, NY) (S2). Sample 3 was islets co-cultured with MSCs with cell-to-cell contact (S3). Sample 4 was MSCs alone as a negative control (S4).

Total RNA was extracted from islets using Isogen (Nippon Gene, Toyama, Japan) with acid guanidinium-phenol-chloroform (the AGPC method), and antisense RNA (aRNA) was synthesized from the obtained 500 ng RNA (Amino Allyl MessageAmp II aRNA Amplification Kit, Ambion, Austin, TX). The synthesized aRNA was labeled with either Cy3 or Cy5 using the Cy3 and Cy5 Mono-Reactive Dye Pack (GE Healthcare, Buckinghamshire, UK). Microarray hybridization was performed with the two-color method. We used two microarray slides of the Agilent G4122F Whole Mouse Genome Microarray 4x44K (Agilent Technologies, Colorado Springs, CO), which contained probes for 21,262 genes. S2/S1 was hybridized on block 1 (B1) of the microarray slide, S3/S1 was hybridized on block 2 (B2), S4/S2 was hybridized on block 3 (B3), and S4/S3 was hybridized on block 4 (B4). The images of the microarray slides were scanned with the GenePix 4000B scanner (Axon Instruments, Ontario, Canada). In the B1 and B2 setting, genes whose expression was significantly increased or decreased upon co-culture with MSCs were considered candidates. In the B3 and B4 setting, it was confirmed that these expression changes were not caused by MSC contamination. A threshold of twofold was chosen for genes that were significantly up- or downregulated.

### Quantitative reverse transcription-polymerase chain reaction (qPCR)

We performed a qPCR assay for the certification and quantification of the expression of eight genes that have biological significance with respect to islet function and were classed as differentially expressed by our microarray analysis. The expression of these eight genes was not different between islets cultured in contact with MSCs versus those cultured with non-contacted MSCs. Therefore, islets were co-cultured with MSCs in a transwell in order to prevent contamination with MSCs themselves.

The primers are shown in Table 1. We extracted total RNAs of S1 and S2 using the acid guanidinium-phenol-chloroform (AGPC method), and the cDNA was synthesized from the RNA using the PrimeScript RT reagent kit (Takara Bio). The qPCR analysis was carried out using Fast Start DNA Master SYBR Green I (Light Cycler; Roche, Mannheim, Germany) with the Light Cycler 1.5 system (Roche). Twenty-five nanograms cDNA per 15 μL was reacted under the following thermal conditions: 95°C for 10 min followed by 45 cycles at 95°C for 10 s, 60°C for 7 s, and 70°C for 10 s. Acquired data were normalized using glyceraldehyde 3-phosphate dehydrogenase (GAPDH) as the reference (28).

**Table 1:** The primer setting for qPCR.

	Forward	Reverse
**Sult1c2**	5’-CTCGATTCTAACTCTGAAATAACTACC-3’	5’-AGATGATCCCTTCTATTTGTATGC-3’
***Trpm5***	5’-CTAGTGAAAGGCATCTCCG-3’	5’-AGGACCTGGATGGTCATTTA-3’
***Rab37***	5’-AGAGCAAAGTCAGCACTCTA-3’	5’-CTCCTCCCTGATCTTCCTATG-3’
***Xaf1***	5’-TAATTGTTAGAAGGGCAATCGTCATA-3’	5’-GATAGTTCAAGATGGCCCTG-3’
***Nr4a2***	5’-AATAGGTGGGCACAAGTATCA-3’	5’-GGAATGTTGGGCGGTGTTA-3’
***Ttyh1***	5’-CAACATGATGCTCTGGGTTAC-3’	5’-TATTCTGAACTAAGCCGCAGAT-3’
***Dapl1***	5’-AGTTCACACGGCACAT-3’	5’-TTTCGAGGTTGCTGAATAATGTAG-3’
***Gstm1***	5’-CTTGCCCAGGAACTCAGAGTAGA-3’	5’-CCGTGCAGACATTGTGGAGA-3’
***GAPDH***	5’-GTGGACCTCATGGCCTACAT-3’	5’-TGTGAGGGAGATGCTCAGTG-3’

### Induction of diabetic recipient mice

Streptozotocin (150 mg/kg body weight [BW]; Sigma-Aldrich) was injected in BALB/c mice (9–12-wk-old male) via the tail vein. Mice whose blood glucose levels consistently exceeded 350 mg/dL in two measurements using a glucometer (Ascencia Breeze 2; Bayer Health Care, Osaka, Japan) were used as diabetic recipient mice.

### Syngenic intramuscular islet transplantation with or without MSCs

Isolated islets (500 islet equivalents: IEQs) were transplanted without MSCs (Group A) or with MSCs (5×10^5^ cells, Group B) into the femur muscle of diabetic recipient mice (n = 15 in each group). The outcome of the transplantation was evaluated in terms of the nonfasting blood glucose level, BW, result of an intraperitoneal glucose tolerance test (IP-GTT), and the cytokine level. IP-GTT (2 g/kg glucose injection) was performed at 28 d post-transplantation. In regard to inflammation, the plasma interleukin (IL)-6 level was also measured with the Mouse IL-6 ELISA kit (Invitrogen, Frederick, MD) at 3 d post-transplantation.

### Histological assessment

The samples were extirpated and fixed at 3 and 28 d post-transplantation and stained by the hematoxylin and eosin (H&E) method, immunohistostaining for insulin and CD31, and the terminal deoxynucleotidyl transferase dUTP nick end labeling (TUNEL) method. Primary antibodies against insulin and CD31 were mouse anti-insulin antibody (Dako, Carpenteria, CA) diluted 1:1000 and goat anti-CD31 (Santa Cruz Biotechnology, Santa Cruz, CA) diluted 1:800. After incubation with biotinylated secondary immunoglobulin (Ig)-G antibody (Nichirei Biosciences, Tokyo), a peroxidase substrate solution containing 3,3′-diaminobenzidine (DAB, brown; Dako) or aminoethylcarbazol (AEC, red for insulin; Dako) was used for visualization. TUNEL staining was done using a Peroxidase In situ Apoptosis Detection kit (Millipore, Billerica, MA) equipped with Discovery XT (Roche). Sections were treated with proteinase K and incubated with TdT enzyme for 60 min at 37°C. After that, the sections were further incubated with streptavidin horseradish peroxidase solution and visualized with DAB.

The quantification of inflammation, apoptosis and neovascularization was performed as follows. The number of infiltrated neutrophils was counted using microscopic observation at 400× magnification, and the number per transplanted islet area (cells per mm^2^) was calculated. The hemorrhagic and necrotic areas were quantified using microscopic observation with 20× magnification, and the area per transplanted site area (%) was calculated. The number of TUNEL-positive cells was counted in the whole transplanted site by using microscopic observation at a 400-fold view, and their number per transplanted area (cells per mm^2^) was calculated. The CD31-positive vessels were quantified using microscopic observation with 400× magnification, and the number per transplanted site area (vessel number per mm^2^) was calculated. (n = 3 areas of islet clusters in each group)

### Statistical analysis

All data are expressed as mean ± standard deviation. Statistical analyses were performed using JMP v.9.0 for Windows (SAS Institute, Cary, NC). A two-way repeated measures analysis of variance (ANOVA) was applied for the comparison of blood glucose levels, the changing rate of BW, and IP-GTT. Student’s unpaired *t*-test was used for evaluation of the other parameters. A significant difference was designated as *p* < 0.05.

## Results

### *In vitro* assessment of islets cultured with MSC

**Fig 1:**
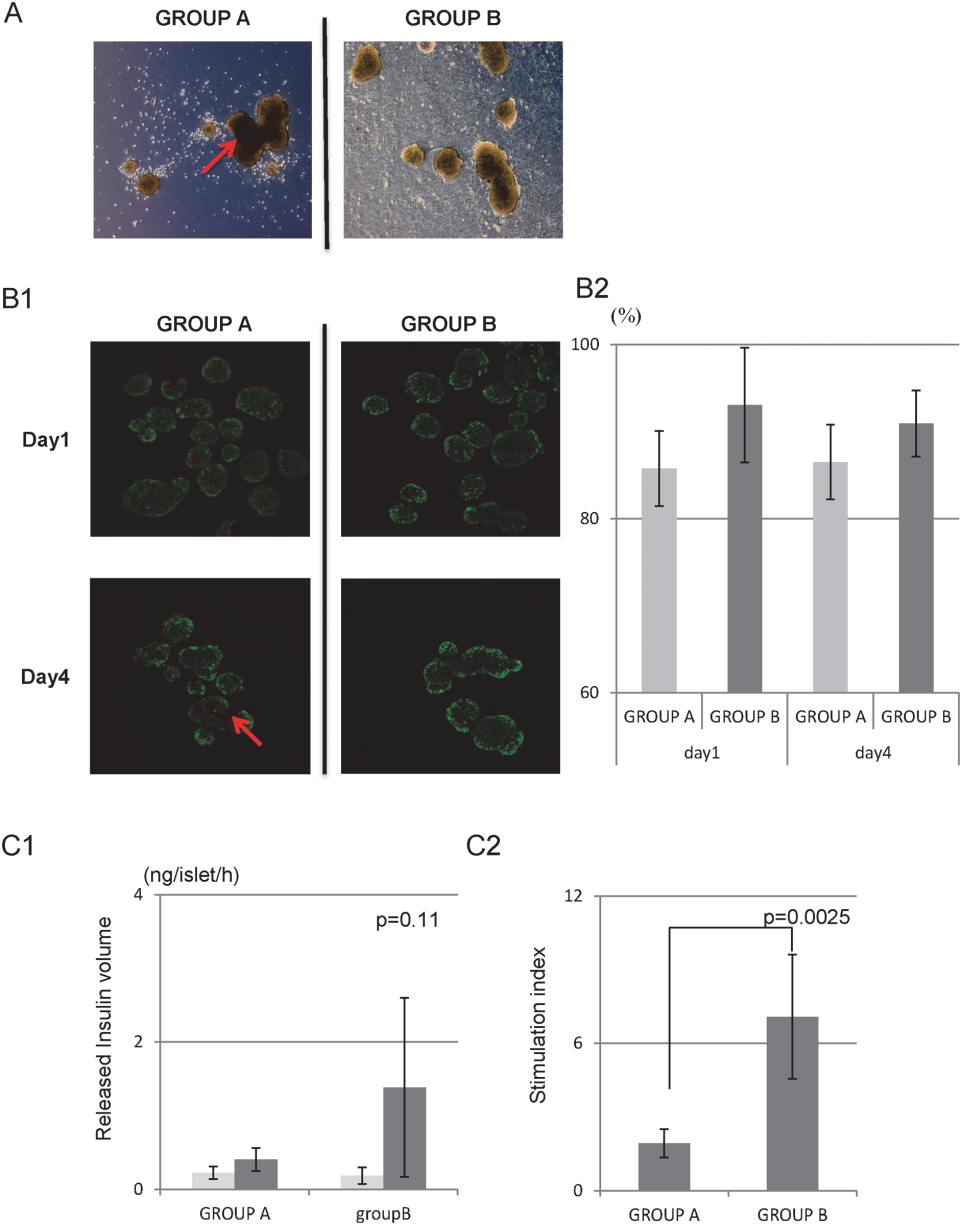
Morphological changes and viability of the islets cultured with or without MSCs. A: Morphological changes of the islets cultured with or without MSC at 4 d after starting the culture. Broken islets and cellular scattering appeared in Group A. Central necrosis (shown as darkness in the center of islets, arrow) was also seen in many residual round-shaped islets. In contrast, there was no cellular scattering caused by islet destruction in Group B. The shapes of the islets were round, and the central colors remained relatively clear. B1: Viability of the 1- and 4-d islets cultured with or without MSCs. Islets were stained with SYTO11 green (green for viable cells) and ethidium bromide (red for dead cells). B2: At 1 d of culture, the viability of Group A was 85.2% and that of Group B was 93% (p = 0.10). At 4 d of culture, the viability of Group A was 86.4% and that of Group B was 90.9% (p = 0.16). C: A glucose stimulation test was performed 4 d after starting the culture. In the resting state (low glucose stimulation), both groups of islets released a small volume of insulin, and there was no significant difference between the two groups (light gray bar). In contrast, the islets in Group B released a higher volume of insulin than those of Group A under high glucose stimulation (dark gray bar) (0.40 ng/islet/h vs. 1.38 ng/islet/h, p = 0.11). The stimulation index in Group B was also significantly higher than that in Group A (1.78 vs. 7.08, p = 0.0025).

Fig. 1A shows the morphological changes of the islets in Groups A and B after 4-d culture. Many broken islets, dispersed cells, and central necrosis were observed in Group A in contrast to Group B. Viability was generally high in both groups, although more viable cells were seen in group B after the 4-d culture (Fig. 1B1). Though there were no significant differences between the two groups, the viability rate of Group B tended to be superior compared to that of Group A (Fig. 1B2). Regarding the glucose response, the islets in Group B released a higher volume of insulin than the islets in Group A upon stimulation (0.40 ± 0.15 ng/islet/h vs. 1.38 ± 1.21 ng/islet/h; p = 0.11; Fig. 1C1). The stimulation index in Group B was significantly higher than that in Group A (1.78 ± 0.59 vs. 7.08 ± 2.53; p = 0.0025; Fig. 1C2). These data revealed that MSC co-culture tended to prevent islet destruction under culture, and preserve insulin-releasing function of cultured islets.

### Assessment of gene expression in islets upon co-culture with syngenic MSCs

**Fig 2:**
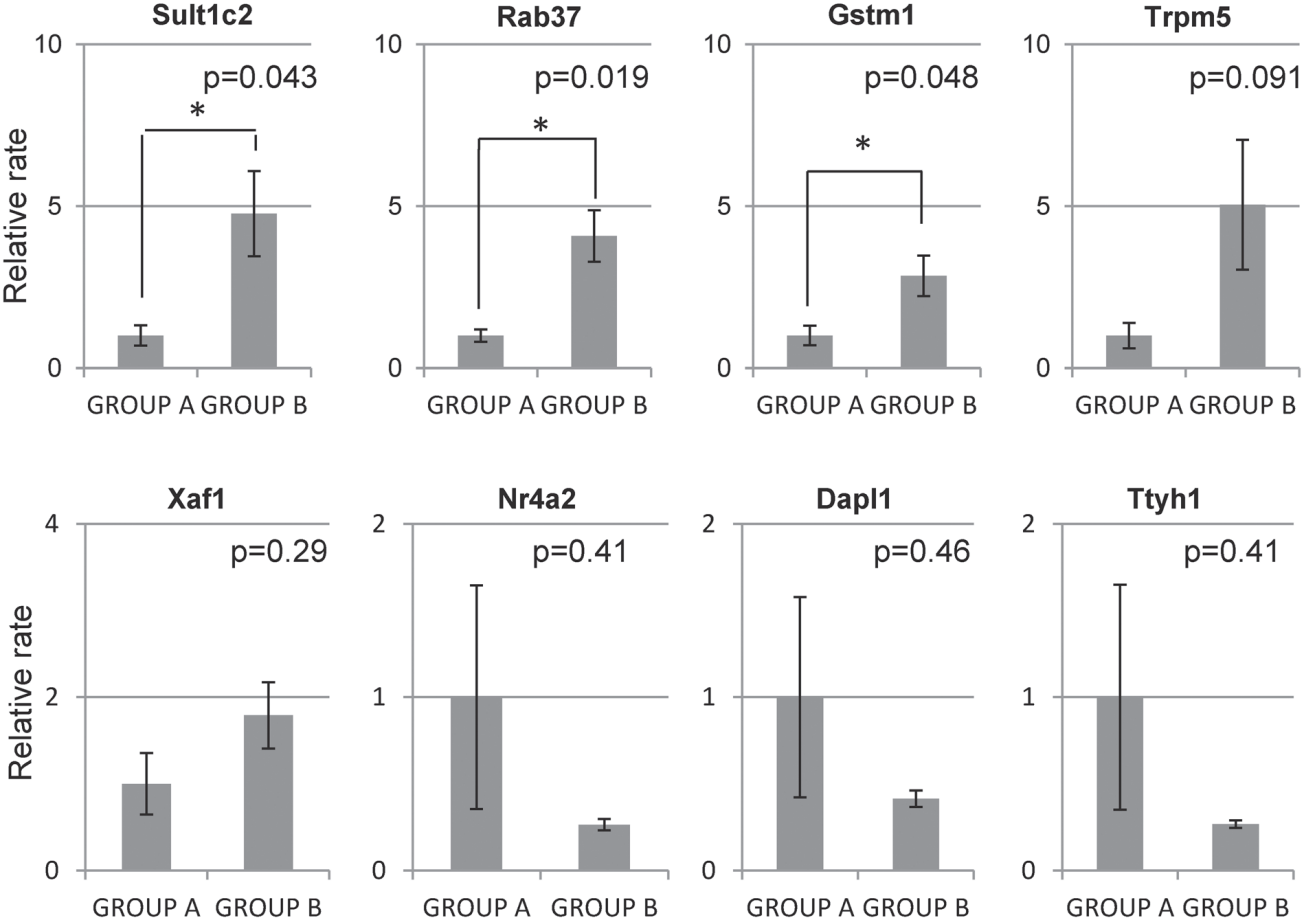
Quantitative PCR. The expressions of selected genes by microarray analysis were quantified. Sult1c2, Gstm1, and Rab37 expressions were significantly increased in Group B compared to Group A.

We performed DNA microarray for detecting upregulated and downregulated genes of islets under MSC co-culturing to clarify the effect of MSCs on the islets. Nineteen upregulated genes and 12 downregulated genes (Table 2 and 3) were detected in the islets cultured with MSCs. This gene expression pattern was similar between islets that were directly co-cultured with MSCs (islets and MSCs were co-cultured in the same space) and those that were separately co-cultured with MSCs using a transwell (islets and MSCs were not in the same space but the islets could receive humoral factors released from MSCs). Hence, this profile was independent of whether the islets were in direct contact with the MSCs. Among the 31 genes, we selected eight that have biological significance with respect to islet function for the quantification using qPCR assay (Table 4). The assay revealed that *Sult1c2* (4.76-fold, p = 0.043), *Gstm1* (2.84-fold, p = 0.048), and *Rab37* (4.07-fold, p = 0.019) were significantly upregulated in Group B compared to Group A (Fig. 2). Rab37 is specifically associated with insulin-containing large dense core granules of pancreatic beta cells, and Gstm1 plays an anti-oxidant role.

**Table 2:** Changes in islet gene expression upon MSC co-culture: Upregulated genes.

Upregulated gene expression		Increasing level
**Sult1c2**	sulfotransferase family	3.595
**Trpm5**	transient receptor potential cation channel	3.520
**Mqarp**	mitochondria localized glutamic acid rich protein	3.453
**Ifit3**	interferon-induced protein with tetratricopeptide repeats3	2.939
**Emid1**	EMI domain containing 1	2.900
**Slc35d3**	solute carrier family 35	2.584
**Ifit1**	interferon-induced protein with tetratricopeptide repeats1	2.939
**Rab37**	member of RAS oncogene family	2.200
**Oasl2**	2′-5′ oligoadenylate synthetase-like 2	2.162
**Dct**	dopachrome tautomerase	2.140
**Gstm1**	glutathione S-transferase mu1	2.105
**Defb1**	defensin beta1	2.103
**Dio1**	deiodinase type1	2.075
**Aldoc**	aldolase C	2.049
**Col3a1**	collagen type1	2.041
**Fam92b**	family with sequence similarity 92	2.022
**Wdr67**	WD repeat domain 67	2.020
**Blnk**	B cell linker	2.015
**Xaf1**	XAIP associated factor1	2.010

**Table 3:** Changes in islet gene expression upon MSC co-culture: Downregulated genes.

Downregulated gene expression		Decreasing level
**Nr4a2**	nuclear receptor subfamily 4	−3.903
**Ttyh1**	twenty homolog 1	−3.141
**Pdyn**	Prodynorphin	−2.894
**Star**	steroidogenic acute regulatory protein	−2.882
**Dapl1**	death associated protein-like 1	−2.780
**Fam46a**	family with sequence similarity 46	−2.599
**Crem**	cAMP responsive element modulator	−2.520
**C2cd4a**	C2 calcium-dependent domain containing 4A	−2.479
**Il11**	interleukin 11	−2.405
**Ccbl2**	cysteine conjugate-beta lyase	−2.291
**Herc4**	hect domain and RLD 4	−2.081
**Prr15**	proline rich 15	−2.019

**Table 4:** Eight genes selected for quantitative PCR analysis.

Up-regulated genes		Down-regulated genes	
**Sult1c2**	Catalyzes several hormones	**Nr4a2**	regulatory factor in T lymphocytes and macrophages
**Trpm5**	related to insulin secretion	**Ttyh1**	twenty 1 homolog 1
**Rab37**	related to insulin secretion	**Dapl1**	relating in apoptosis
**Gstm1**	anti-oxidant stress enzyme		
**Xaf1**	inhibitor of apoptosis		

In summary, co-cultured MSCs induced the islets to express some genes that supported islet function.

### Therapeutic effects of co-transplanted MSCs

**Fig 3:**
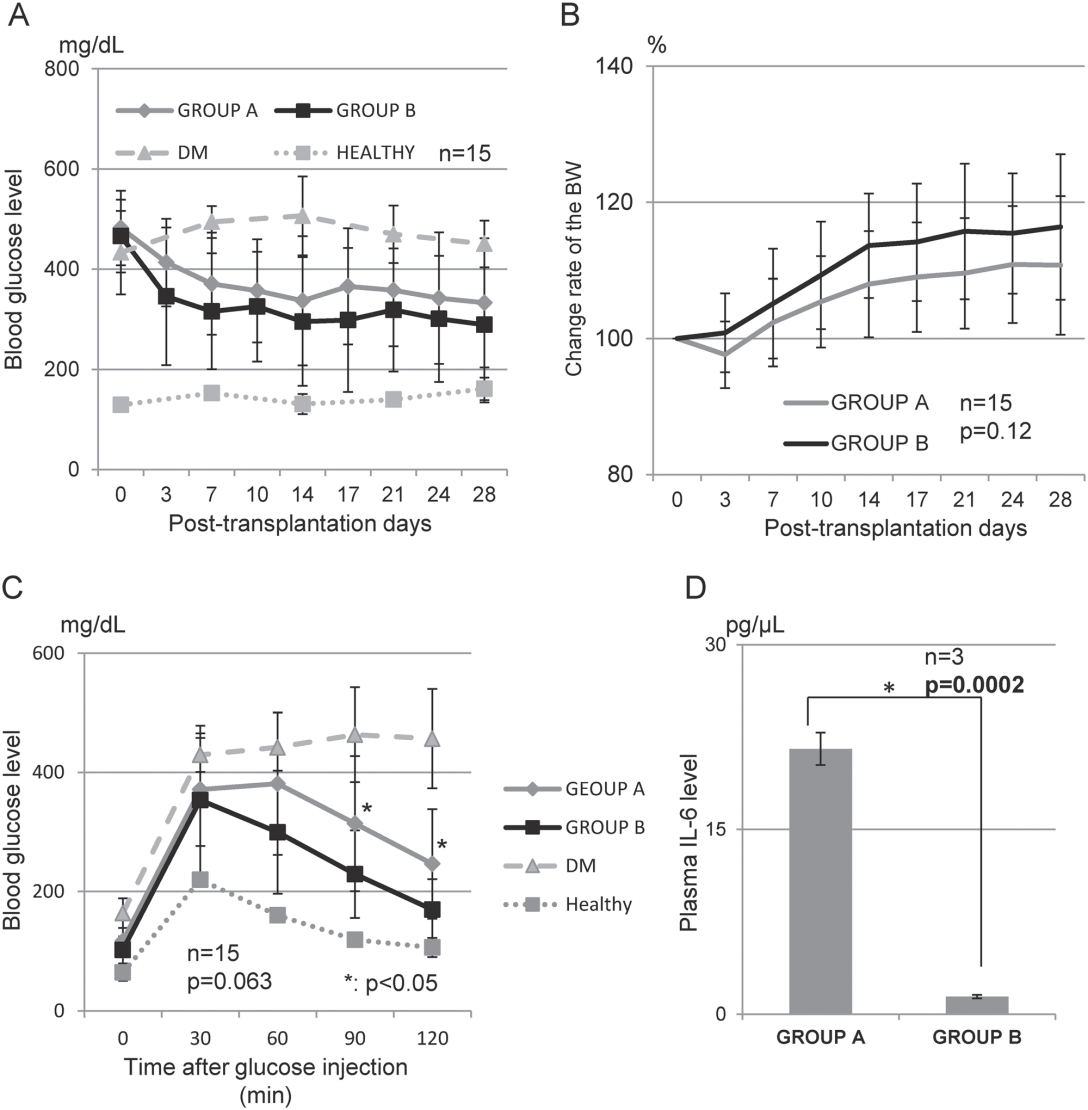
Therapeutic effects of co-transplantation with MSCs. A: Blood glucose level. The blood glucose level was measured after transplantation. The blood glucose level of Group B tended to show improved hyperglycemia more than Group A. B: Body weight (BW) changes. In Group A, the BW temporarily decreased, and then continuously increased. In contrast, the BW in Group B increased continually starting early after transplantation. The changes in Group B tended to be higher than those in Group A. C: IP-GTT. At 28 d post-transplantation, IP-GTT was estimated as a measure of graft function. Although the peak glucose levels in Groups A and B were similar, normoglycemia was achieved faster in Group B. D: Plasma IL-6 level. The plasma IL-6 level at 3 d post-transplantation was significantly lower in Group B than in Group A.

Fig. 3 shows the therapeutic outcomes of islet transplantation without (Group A) and with MSCs (Group B). The glucose level of the diabetic mice was decreased after the intramuscular islet transplantation over time in both Groups A and B (Fig. 3A). Although the therapeutic effects of MSCs were observed in the *in vitro* assessments, little effectiveness was observed in the transplantation model. The blood glucose levels of Group B were consistently lower than those of Group A, but this difference did not reach the level of significance (p = 0.22, Fig. 3A). The BW values temporarily decreased and then increased in Group A, and in Group B, they consistently increased; however, this difference did not reach the level of significance (p = 0.12, Fig. 3B).

The therapeutic effect of MSC co-transplantation was seen in the IP-GTT evaluation. The post-peak level was significantly improved in Group B (314 ±113.5 mg/dL vs. 229.1 ± 73.5 mg/dL, p = 0.026 at 90 min and 246.2 mg/dL vs. 169.5 mg/dL, p = 0.011 at 120 min, Fig. 3C). Although the therapeutic effects of MSCs for treating DM were not prominent in the intramuscular transplantation model, an anti-inflammatory effect was demonstrated. Fig. 3D shows that the plasma IL-6 level at 3 d post-transplantation, and it was significantly lower in Group B than in Group A (21.5 ± 2.3 pg/μL vs. 1.40 ± 1.1 pg/μL, p = 0.0002).

### Histological analysis

**Fig 4:**
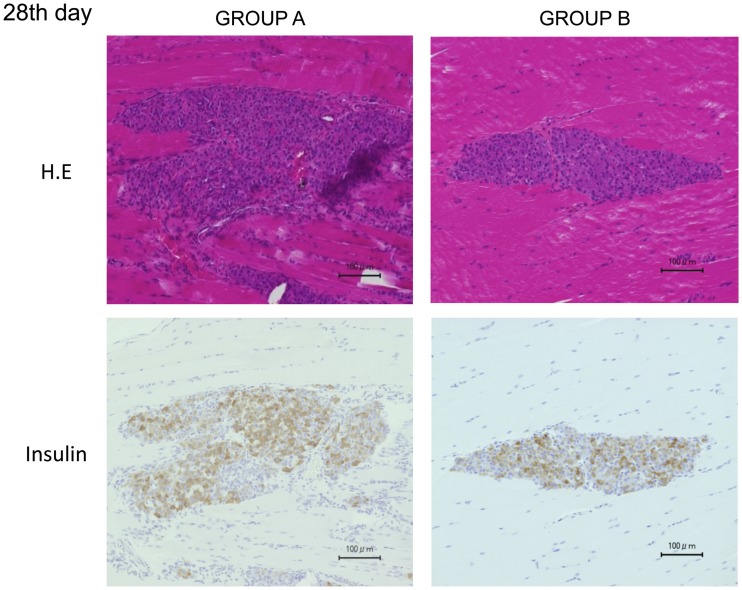
Histological examination of engrafted islets at 28 d post-transplantation. The engrafted islets in both groups were in good morphological condition 28 d post-transplantation. Insulin staining intensity was similar between Groups A and B.

**Fig 5:**
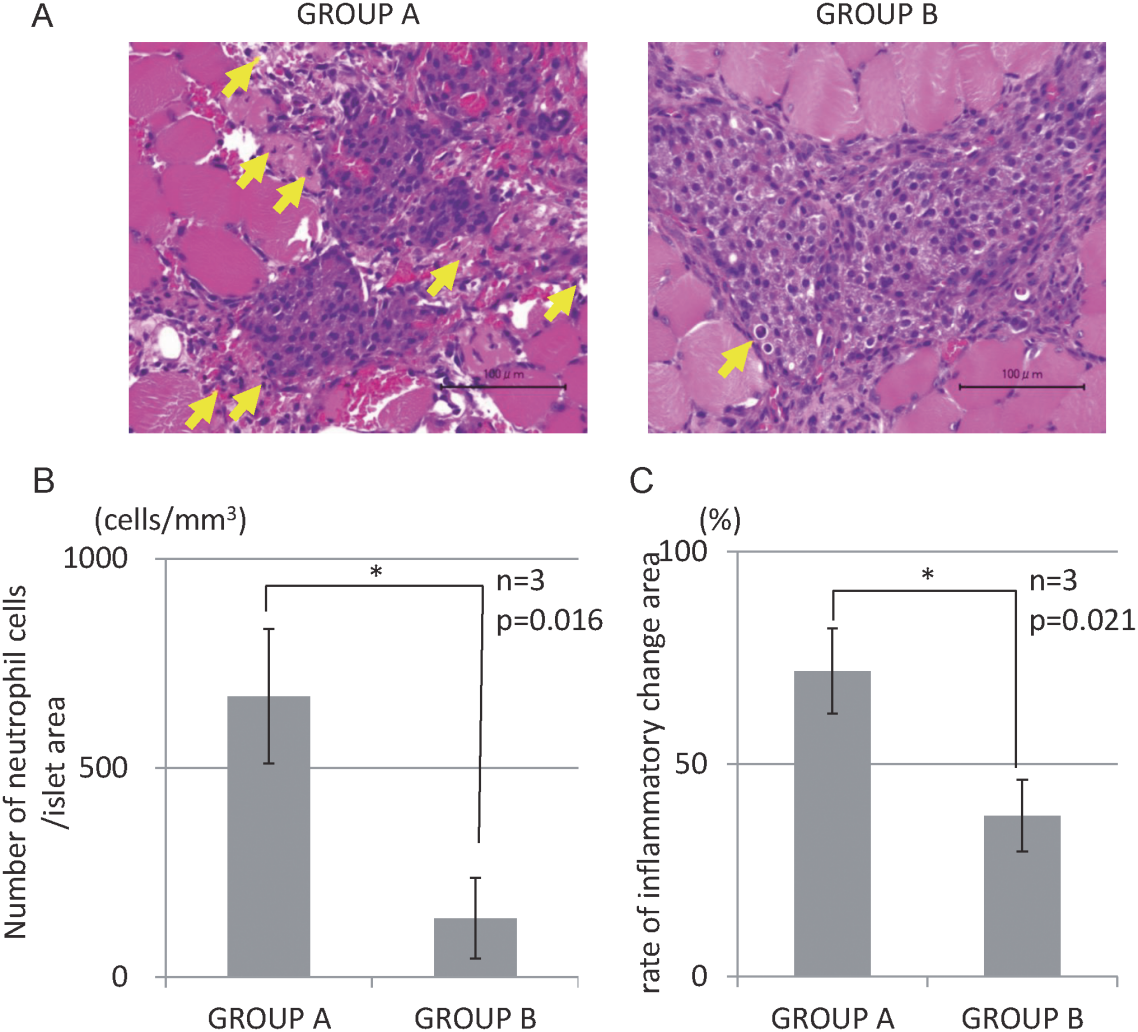
Histological examination for inflammation at the transplant site 3 d post-transplantation. A: More neutrophils (yellow arrows) were seen around transplanted islets in Group A. The amounts of hemorrhage and necrosis around islets in Group A was more extensive than in Group B. B: The number of neutrophils in Group B was significantly lower than that in Group A (p = 0.016). C: Bleeding and necrosis were closely correlated with inflammation. The inflammatory changing area was defined as the bleeding and necrosis area. The rate of inflammatory change at the transplantation site was significantly lower in Group B than in Group A (p = 0.021).

**Fig 6:**
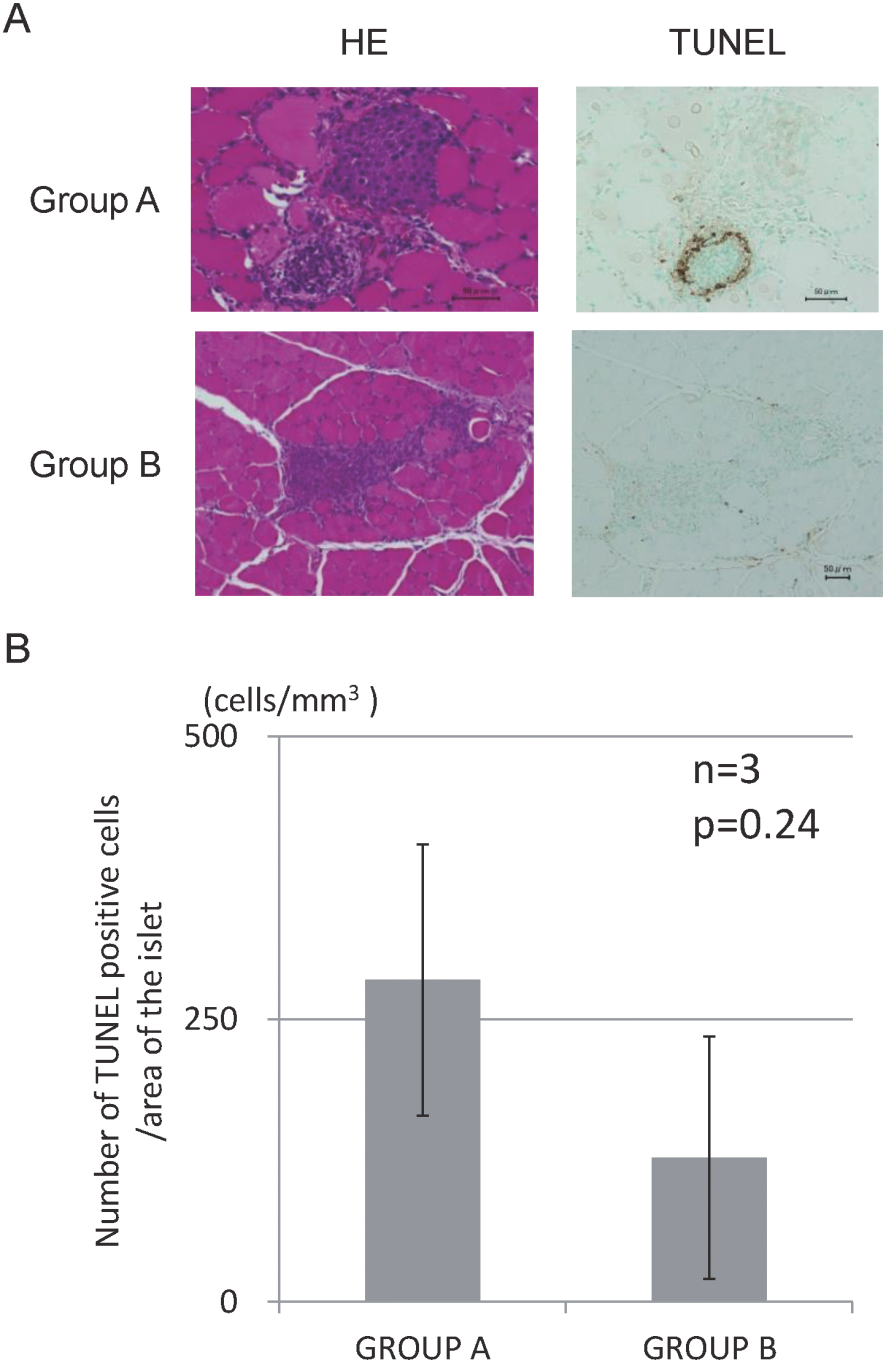
Histological examination for apoptosis 3 d post-transplantation. TUNEL-positive cells were less frequent in Group B than in Group A, but this difference did not reach significance.

**Fig 7:**
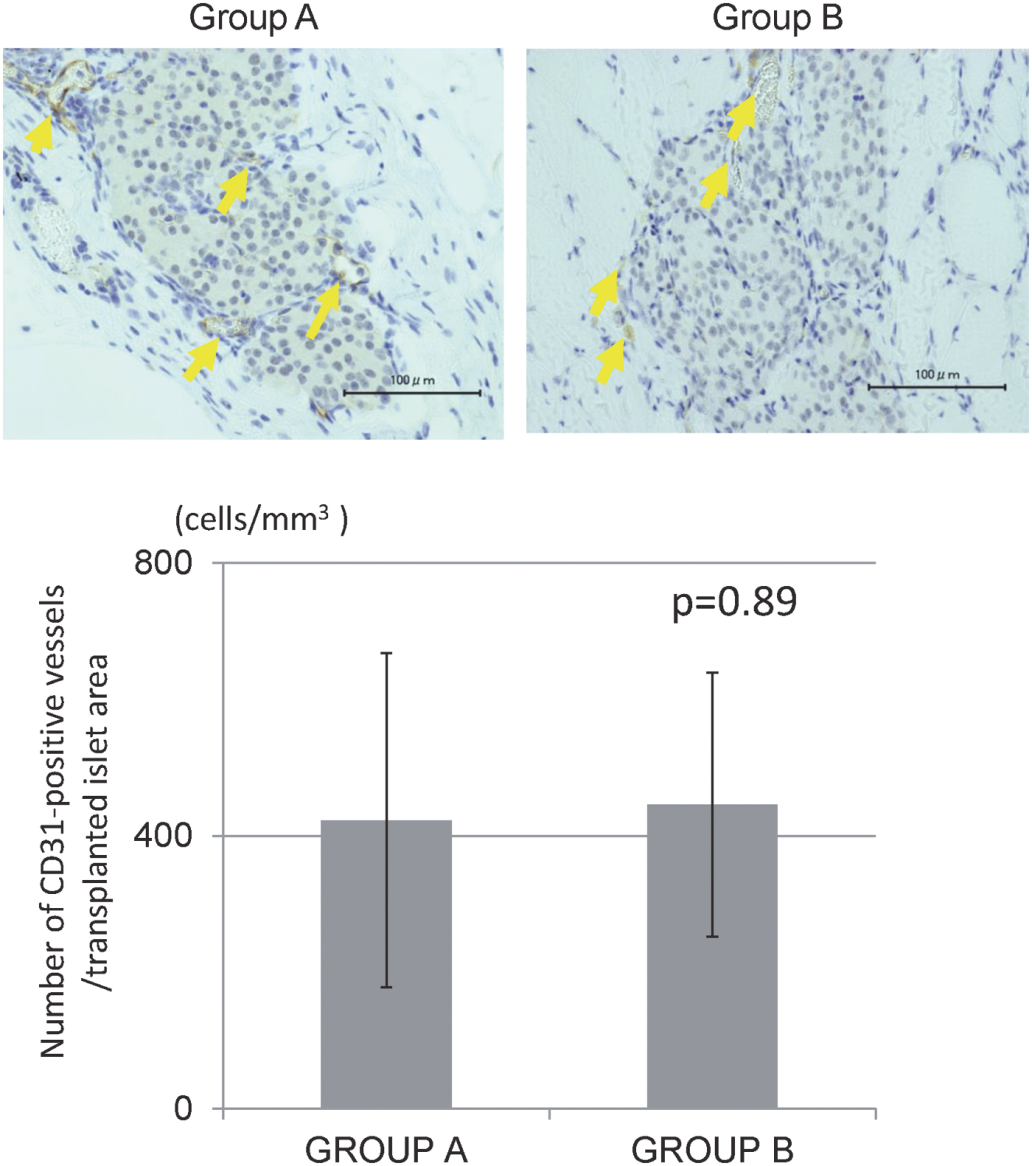
Histological examination for neovascularization at 28 d post-transplantation. CD31 was expressed in blood vessels, and the number of CD31-positive vessels (yellow arrow) was similar in the two groups.

The histological findings revealed that islets engrafted into muscles in both groups displayed good morphology up to 28 d after transplantation. The staining intensity of insulin was similar between the two groups (Fig. 4). There were few inflammatory cells, hemorrhages, and necrosis in the transplantation site of Group B, whereas many inflammatory changes were observed in Group A at 3 d after transplantation (Fig. 5A). The proportions of inflammatory cells and inflammatory area of Group B were significantly less than those of Group A (671.1 cells/mm^3^ vs. 140.1 cells/mm^3^, p = 0.016, Fig. 5B, and 72.0% vs. 37.9%, p = 0.021, Fig. 5C). Regarding apoptosis, more TUNEL-positive cells in islets were observed in Group A (284.7 cells/mm^3^) than in Group B (127.4 cells/mm^3^), but there was no significant difference (p = 0.24, Fig. 6). The numbers of CD31-positive vessels around the islets were almost the same between Groups A and B (422.7 ± 245.2/mm^2^ vs. 445.4 ± 193.3, respectively; p = 0.89, Fig. 7). At least, the anti-inflammatory effect of MSCs was prominent not only in terms of the serum IL-6 level but also in the histological inflammatory changes.

## Discussion

This study reproduced the results of previous reports that the intramuscular transplantation of islets has a therapeutic effect on diabetic mice. We were able to clarify that MSCs maintained the morphological changes in co-cultured islets and conserved the islet function. MSCs stimulated islets to express some genes related to the process of insulin release and anti-oxidant function. However, while the utility of the MSCs was clear in the *in vitro* examinations, the transplant efficacy of intramuscular islet transplantation was not markedly enhanced by MSC co-transplantation. The data also indicate that the co-transplantation of MSCs prevented an early inflammatory reaction from islets, but could not promote neovascularization between the islet and muscle.

The present study revealed that inflammation after transplantation was significantly suppressed by MSC co-transplantation. The level of the pro-inflammatory cytokine IL-6 and immune reaction as evidenced by bleeding and neutrophil infiltration at the early stage of transplantation were significantly reduced by MSC co-transplantation. While the mechanism of the anti-inflammatory effect of MSCs is not clear, some reports supported our data. For example, both Meng and Tu revealed that MSC transplantation reduced inflammation associated with pancreatitis [30,31]. In inflammatory orthopedic diseases, MSC transplantation could reduce the severity of arthritis in murine models and modulate the expression of inflammatory cytokines [32].

One of the most important factors for the success of islet transplantation is neovascularization [25]. Though we anticipated that neovascularization would be promoted by vascular endothelial growth factor (VEGF) secretion of MSCs in the intramuscular transplantation, the number of blood vessels around the islet at 28 d was almost the same. With regard to neovascularization, Svensson et al. reported that intramuscular transplantation was more effective at establishing a new vascular network than transplantation into the subcapsule of the kidney, and that the oxygen tension of islet grafts was higher in the intramuscular site [33].

However, the muscle surrounding engrafted islets possibly consumed more oxygen than other sites. Otherwise, oxygen consumption might be increased by co-transplantation of MSCs. Indeed, muscle contraction may be harmful to transplanted islets. Regarding the selection of muscle as a transplantation site, an optimal muscle should be selected while excluding the femur muscle. Sakata showed that when bone marrow cells were co-transplanted with islets into the renal subcapsule in a murine model, the high VEGF secretion in the transplantation site resulted in reduced apoptosis of islets and improved glycemic control by inducing neovascularization [25]. Since the anti-inflammatory effect alone couldn’t reduce apoptosis, the improvement of glycemic control, early nutrition and oxygen supply might be essential for achieving successful intramuscular islet transplantation.

Our findings also revealed that MSCs had many biological impacts on the co-cultured islets themselves. For example, the insulin stimulation index was significantly improved by co-culture with MSCs. In addition, molecular profiling revealed that MSCs increased the expression of some islet related genes upon co-culture with humoral factors without cell-to-cell contact. In particular, *Rab37*, *Gstm1*, and *Sult1c2* were significantly increased. Rab37 belongs to a subclass of Rab GTPases that regulate exocytosis, and it is highly enriched in beta cells [34,35]. Rab37 is specifically associated with insulin-containing large dense core granules of pancreatic beta cells [36]. *Gstm1* encodes a member of the glutathione S-transferase family (which is comprised of phase II detoxification enzymes [37]) and plays an anti-oxidant role.

Isolated islets can be damaged by oxidant stress during the isolation process. Therefore, increasing anti-oxidant enzyme function in the co-cultured islets might prevent damage and improve the islet function. *Sult1c2* encodes one of the sulfotransferase enzymes which catalyze the synthesis of many hormones [38]. Sult1c2 may support islet function, although the underlying mechanisms are unclear. The *Rab37* gene has important roles in insulin secretion. And thus Rab37, Gstm1 and Sult1c2 may have contributed to improved islet function in the *in vitro* study. These expressed genes might become a target of treatments to improve the outcome of islet transplantation.

It has been thought that MSCs have lower oncogenic potential in comparison with embryonic stem (ES) and induced pluripotent stem (iPS) cells [39,40], but recently some research groups have clarified the oncogenicity of MSCs. Fiorina and colleague found tumor formation in non-obese diabetic (NOD)-MSC-treated mice [41,42]. Miura and colleagues revealed that murine MSCs became malignant fibrosarcoma cells after numerous passages [43]. These phenomena indicate that transplanted MSCs can acquire oncogenicity during long-term follow-up, and therefore intensive postoperative observation is necessary for MSC-transplanted patients in the clinical setting, the same as with ES and iPS cells.

In terms of the transplantation site, the advantage of intramuscular injection is still promising. Both groups showed some significant therapeutic effects on DM mice, although the blood glucose level couldn’t be completely reduced. Since this study showed that MSCs have an anti-inflammatory effect, the repetitive injection of islets with MSCs may be preferable to an islets-alone injection. The preconditioning of MSCs and the MSC / islet ratio remain to be examined. Furthermore, the extracellular matrix, which has a cellular protective effect for transplanted cells [44], might be a good device to improve the effect of MSC co-transplantation.

In summary, MSC co-transplantation had a positive effect on islet function *in vitro* and suppressed the inflammatory reaction at the transplant sites. Intramuscular transplantation of islets with or without MSCs had a therapeutic effect on diabetic syngenic mice, whereas the augmentation by the co-transplantation was limited. Further investigations for the promotion of neovascularization and the expression of the genes that support islet function may validate the utility of MSC co-transplantation for intramuscular islet transplantation.

## Supporting Information

S1 Fig The characterization of pluripotency and the profile of MSCs. A: Osteogenic differentiation. The bone matrix was stained red by Alizarin Red O (right) to confirm the osteogenic differentiation potential. B: Adipogenic differentiation. Adipocyte oil droplets were stained red by Oil Red O (right) to confirm the adipocyte differentiation potential. C: MSC surface markers. All of the MSC-positive markers (CD29, CD44, CD73, CD105, CD106, and Sca1) were detected, and negative markers (CD11b and CD45) were not present.
(TIF)

## References

[1] Kemp CB, Knight MJ, Scharp DW, Lacy PE, Ballinger WF (1973) Transplantation of isolated pancreatic islets into the portal vein of diabetic rats. Nature 244: 447 4200461 10.1038/244447a0

[2] Sakata N, Aoki T, Yoshimatsu G, Tsuchiya H, Hata T, et al (2013) Strategy for clinical setting in intramuscular and subcutaneous islet transplantation. Diabetes Metab Res Rev 1:1–10. 10.1002/dmrr.2463 24000195

[3] Bennet W, Groth CG, Larsson R, Nilsson B, Korsgren O (2000) Isolated human islets trigger an instant blood mediated inflammatory reaction: implications for intraportal islet transplantation as a treatment for patients with type 1 diabetes. Ups J Med Sci 105: 125–133 11095109 10.1517/03009734000000059

[4] Bottino R, Fernandez LA, Ricordi C, Lehmann R, Tsan MF, et al (1998) Transplantation of allogeneic islets of Langerhans in the rat liver: effects of macrophage depletion on graft survival and microenvironment activation. Diabetes 47: 316–323 9519734 10.2337/diabetes.47.3.316

[5] Ishiyama K, Rawson J, Omori K, Mullen Y (2011) Liver natural killer cells play a role in the destruction of islets after intraportal transplantation. Transplantation 91: 952–960 10.1097/TP.0b013e3182139dc1 21389902

[6] Leitao CB, Bernetti K, Tharavanij T, Cure P, Lauriola V, et al (2010) Lipotoxicity and decreased islet graft survival. Diabetes Care 33: 658–660 10.2337/dc09-1387 20009097 PMC2827526

[7] Lee Y, Ravazzola M, Park BH, Bashmakov YK, Orci L, et al (2007) Metabolic mechanisms of failure of intraportally transplanted pancreatic beta-cells in rats: role of lipotoxicity and prevention by leptin. Diabetes 56: 2295–2301 17563069 10.2337/db07-0460

[8] Sakata N, Hayes P, Tan A, Chan NK, Mace J, et al (2009) MRI assessment of ischemic liver after intraportal islet transplantation. Transplantation 87: 825–830 10.1097/TP.0b013e318199c7d2 19300184 PMC2779521

[9] Sakata N, Obenaus A, Chan N, Mace J, Chinnock R, et al (2009) Factors affecting islet graft embolization in the liver of diabetic mice. Islets 1: 26–33 10.4161/isl.1.1.8563 21084846

[10] Kawahara T, Kin T, Kashkoush S, Gala-Lopez B, Bigam DL, et al (2011) Portal vein thrombosis is a potentially preventable complication in clinical islet transplantation. Am J Transplant 12: 2700–7. 10.1111/j.1600-6143.2011.03717.x 21883914 PMC3226916

[11] Sakata N, Goto M, Motoi F, Hayashi H, Nakagawa K, et al (2013) Clinical experiences in the treatment of pancreatic arteriovenous malformation by total pancreatectomy with islet autotransplantation. Transplantation 96: e38–e40 10.1097/TP.0b013e3182a01333 23995868

[12] Fiorina P, Secchi A (2007) Pancreatic islet cell transplant for treatment of deabetes. Endocrinol Metab Clin North Am 36: 999–1013 17983933 10.1016/j.ecl.2007.07.004

[13] Sakata N, Tan A, Chan N, Obenaus A, Mace J, et al (2009) Efficacy comparison between intraportal and subcapsular islet transplants in a murine diabetic model. Transplant Proc 41: 346–349 10.1016/j.transproceed.2008.08.155 19249553 PMC2684877

[14] Kin T, Korbutt GS, Rajotte RV (2003) Survival and metabolic function of syngeneic rat islet grafts transplanted in the omental pouch. Am J Transplant. 3: 281–285 12614282 10.1034/j.1600-6143.2003.00049.x

[15] Maffi P, Balzano G, Ponzoni M, Nano R, Sordi V, et al (2013) Autologous pancreatic islet transplantation in human bone marrow. Diabetes 10: 3523–31. 10.2337/db13-0465 PMC3781459 23733196

[16] Stagner JI, Rilo HL, White KK (2007) The pancreas as an islet transplantation site. Confirmation in a syngeneic rodent and canine autotransplant model. JOP 8: 628–636 17873472

[17] Mojibian M, Harder B, Hurlburt A, Bruin JE, Asadi A, et al (2013) Implanted islets in the anterior chamber of the eye are prone to autoimmune attack in a mouse model of diabetes. Diabetologia 56: 2213–2221 10.1007/s00125-013-3004-z 23933952

[18] Kim HI, Yu JE, Park CG, Kim SJ (2010) Comparison of four pancreatic islet implantation sites. J Korean Med Sci 25: 203–210 10.3346/jkms.2010.25.2.203 20119571 PMC2811285

[19] Lund T, Korsgren O, Aursnes IA, Scholz H, Foss A (2010) Sustained reversal of diabetes following islet transplantation to striated musculature in the rat. J Surg Res 160: 145–154 10.1016/j.jss.2008.11.009 19394966

[20] Rajab A (2010) Islet transplantation: alternative sites. Curr Diab Rep 10: 332–337 10.1007/s11892-010-0130-6 20665132

[21] Brunt KR, Hall SR, Ward CA, Melo LG (2007) Endothelial progenitor cell and mesenchymal stem cell isolation, characterization, viral transduction. Methods Mol Med 139: 197–210 18287673 10.1007/978-1-59745-571-8_12

[22] Hematti P, Kim J, Stein AP, Kaufman D (2013) Potential role of mesenchymal stromal cells in pancreatic islet transplantation. Transplant Rev (Orlando) 27: 21–29 10.1016/j.trre.2012.11.003 23290684

[23] Ito T, Itakura S, Todorov I, Rawson J, Asari S, et al (2010) Mesenchymal stem cell and islet co-transplantation promotes graft revascularization and function. Transplantation 89: 1438–1445 20568673 10.1097/tp.0b013e3181db09c4

[24] Sakata N, Goto M, Yoshimatsu G, Egawa S, Unno M (2011) Utility of co-transplanting mesenchymal stem cells in islet transplantation. World J Gastroenterol 17: 5150–5155 10.3748/wjg.v17.i47.5150 22215938 PMC3243880

[25] Sakata N, Chan NK, Chrisler J, Obenaus A, Hathout E (2010) Bone marrow cell cotransplantation with islets improves their vascularization and function. Transplantation 89: 686–693 10.1097/TP.0b013e3181cb3e8d 20101199 PMC2844476

[26] Park KS, Kim YS, Kim JH, Choi B, Kim SH, et al (2010) Trophic molecules derived from human mesenchymal stem cells enhance survival, function, and angiogenesis of isolated islets after transplantation. Transplantation 89: 509–517 10.1097/TP.0b013e3181c7dc99 20125064

[27] Yoshimatsu G, Sakata N, Tsuchiya H, Ishida M, Motoi F, et al (2013) Development of polyvinyl alcohol bioartificial pancreas with rat islets and mesenchymal stem cells. Transplant P 45: 1875–1880 10.1016/j.transproceed.2013.01.043 23769061

[28] Gotoh M, Maki T, Kiyoizumi T, Satomi S, Monaco AP (1985) An improved method for isolation of mouse pancreatic islets. Transplantation 40: 437–438 2996187 10.1097/00007890-198510000-00018

[29] Sakata N, Egawa S, Sumi S, Unno M (2008) Optimization of glucose level to determine the stimulation index of isolated rat islets. Pancreas 36: 417–423 10.1097/MPA.0b013e31815ccad2 18437089

[30] Meng HB, Gong J, Zhou B, Hua J, Yao L, et al (2013) Therapeutic effect of human umbilical cord-derived mesenchymal stem cells in rat severe acute pancreatitis. Int J Clin Exp Pathol 6: 2703–2712 24294357 PMC3843251

[31] Tu XH, Song JX, Xue XJ, Guo XW, Ma YX, et al (2012) Role of bone marrow-derived mesenchymal stem cells in a rat model of severe acute pancreatitis. World J Gastroenterol 18: 2270–2279 10.3748/wjg.v18.i18.2270 22611322 PMC3351779

[32] Augello A, Tasso R, Negrini SM, Cancedda R, Pennesi G (2007) Cell Therapy using allogenic bone marrow mesenchymal stem cells prevents tissue damage in collagen-induced arthritis. Arthritis Rheum 56: 1175–1186 17393437 10.1002/art.22511

[33] Svensson J, Lau J, Sandberg M, Carlsson PO (2011) High vascular density and oxygenation of pancreatic islets transplanted in clusters into striated muscle. Cell Transplant 20: 783–788 10.3727/096368910X536527 21054943

[34] Brunner Y, Coute Y, Iezzi M, Ftoi M, Fukuda M, et al (2007) Proteomics analysis of insulin secretory granules. Mol Cell Proteomics 6: 1007–1017 17317658 10.1074/mcp.M600443-MCP200

[35] Petyuk VA, Qian WJ, Hinault C, Gritsenko MA, Singhal M, et al (2008) Characterization of the mouse pancreatic islet proteome and comparative analysis with other mouse tissues. J Proteome Res 7: 3114–3126 10.1021/pr800205b 18570455 PMC2749725

[36] Ljubicic S, Bezzi P, Brajkovic S, Nesca V, Guay C, et al (2013) The GTPase Rab37 participates in the control of insulin exocytosis. PLoS One 8: e68255 23826383 10.1371/journal.pone.0068255 PMC3694898

[37] Dadbinpour A, Sheikhha MH, Darbouy M, Afkhami-Ardekani M (2013) Investigating GSTT1 and GSTM1 null genotype as the risk factor of diabetes type 2 retinopathy. J Diabetes Metab Disord 12: 48 10.1186/2251-6581-12-48 24355557 PMC7968338

[38] Paul P, Suwan J, Liu J, Dordick JS, Linhardt RJ (2012) Recent advances in sulfotransferase enzyme activity assays. Anal Bioanal Chem 403: 1491–1500 10.1007/s00216-012-5944-4 22526635 PMC3425364

[39] Hovatta O, Jaconi M, Töhönen V, Bena F, Gimelli S, et al (2010) A teratocarcinoma-like human embryonic stem cell (hESC) line and four hESC lines reveal potentially oncogenic genomic changes. PloS One 5: e10263 10.1371/journal.pone.0010263 20428235 PMC2859053

[40] Griscelli F, FeRaud O, Oudrhiri N, Gobbo E, Casal I, et al (2012) Malignant germ cell-like tumors, expressing Ki-1 antigen (CD30), are revealed during in vivo differentiation of partially reprogramed human-induced pluripotent stem cells. Am J pathol 180: 2084–2096 10.1016/j.ajpath.2012.01.011 22425713

[41] Fiorina P, Jurewicz M, Augello A, Vergani A, Dada S, et al (2009) Immunomodulatory function of bone marrow-derived mesenchymal stem cells in experimental autoimmune type 1 diabetes. J Immunol 183: 993–1004 10.4049/jimmunol.0900803 19561093 PMC3895445

[42] Fiorina P, Voltarelli, Zavazava N (2011) Immunological applications of stem cells in type 1 diabetes. Endocr Rev 32: 725–754 10.1210/er.2011-0008 21862682 PMC3591677

[43] Miura M, Miura Y, Padilla-Nash HM, Molinolo AA, Fu B, et al (2006) Accumulated chromosomal instability in murine bone marrow mesenchymal stem cells leads to malignant transformation. Stem Cells 24:1095–1103 16282438 10.1634/stemcells.2005-0403

[44] Beatti GM, Leibowitz G, Lopez AD, Levine F, Hayek A, et al (2000) Protection from cell death in cultured human fetal pancreatic cells. Cell Transplant 9: 431–438. 10972342 10.1177/096368970000900314

