# Supplementary figures and images for: The Co-Transplantation of Bone Marrow Derived Mesenchymal Stem Cells Reduced Inflammation in Intramuscular Islet Transplantation
Source: PLoS One. 2015 Feb 13;10(2):e0117561. doi: 10.1371/journal.pone.0117561 (PMC4332659; doi:10.1371/journal.pone.0117561)

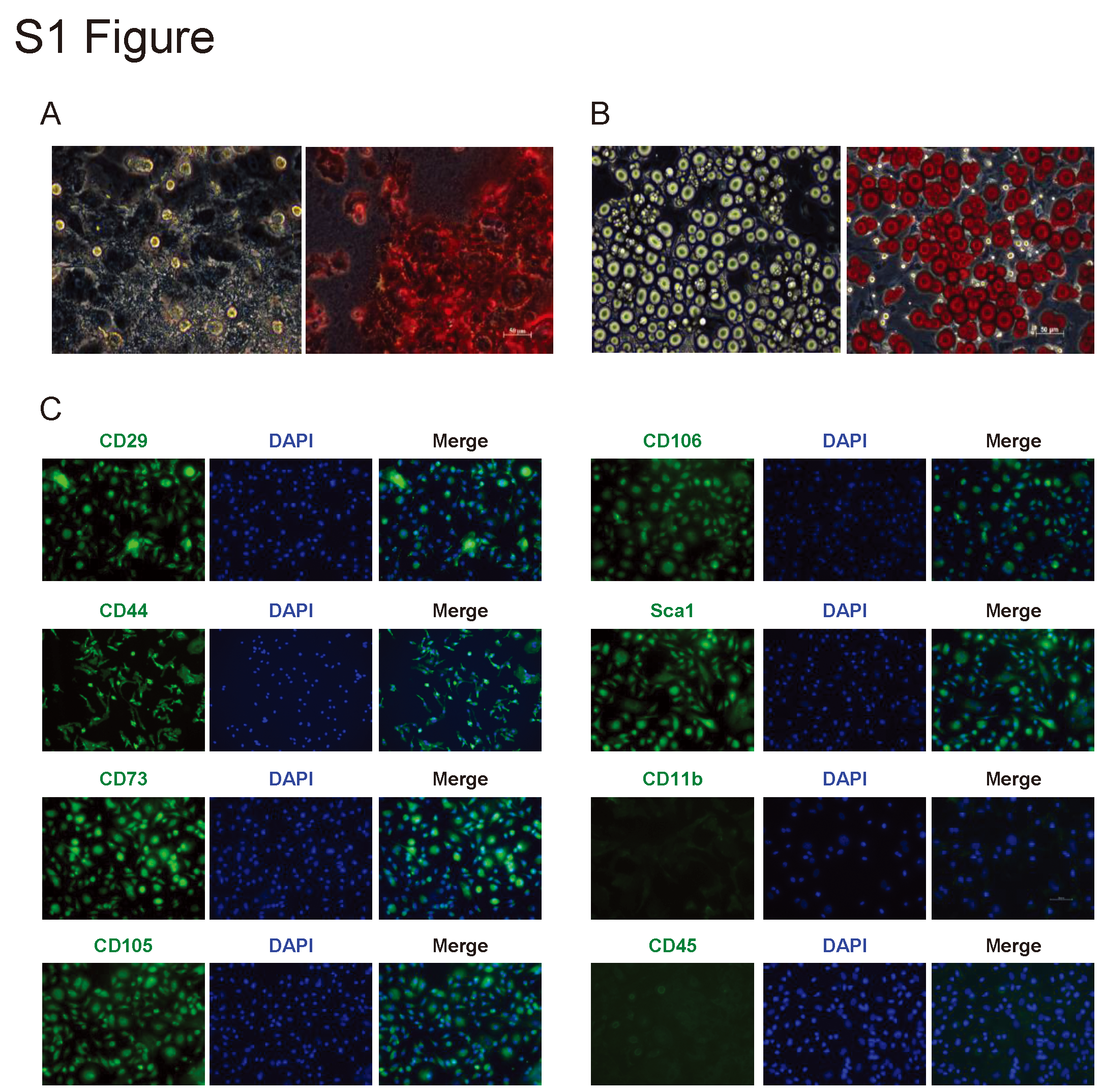

Supplement: S1 Fig — A: Osteogenic differentiation. The bone matrix was stained red by Alizarin Red O (right) to confirm the osteogenic differentiation potential. B: Adipogenic differentiation. Adipocyte oil droplets were stained red by Oil Red O (right) to confirm the adipocyte differentiation potential. C: MSC surface markers. All of the MSC-positive markers (CD29, CD44, CD73, CD105, CD106, and Sca1) were detected, and negative markers (CD11b and CD45) were not present. (TIF) [file pone.0117561.s001.tif]
